# *TRMT61B* rs4563180 G>C variant reduces hepatoblastoma risk: a case-control study of seven medical centers

**DOI:** 10.18632/aging.204926

**Published:** 2023-08-01

**Authors:** Dingyuan Zeng, Jinhong Zhu, Jingjing Li, Fan Liao, Zhonghua Yang, Yong Li, Jiao Zhang, Jiwen Cheng, Suhong Li, Li Li, Jing He

**Affiliations:** 1 Department of Gynecology and Obstetrics, Guangxi Clinical Research Center for Obstetrics and Gynecology, Liuzhou Key Laboratory of Gynecologic Oncology, Liuzhou Hospital, Guangzhou Women and Children’s Medical Center, Liuzhou 545616, Guangxi, China; 2 Department of Clinical Laboratory, Biobank, Harbin Medical University Cancer Hospital, Harbin 150040, Heilongjiang, China; 3 Department of Pediatric Surgery, Guangzhou Institute of Pediatrics, Guangdong Provincial Key Laboratory of Research in Structural Birth Defect Disease, Guangzhou Women and Children’s Medical Center, Guangzhou Medical University, Guangzhou 510623, Guangdong, China; 4 Department of Pediatric Surgery, Shengjing Hospital of China Medical University, Shenyang 110004, Liaoning, China; 5 Department of Pediatric Surgery, Hunan Children’s Hospital, Changsha 410004, Hunan, China; 6 Department of Pediatric Surgery, The First Affiliated Hospital of Zhengzhou University, Zhengzhou 450052, Henan, China; 7 Department of Pediatric Surgery, The Second Affiliated Hospital of Xi’an Jiaotong University, Xi’an 710004, Shaanxi, China; 8 Department of Pathology, Children Hospital and Women Health Center of Shanxi, Taiyuan 030013, Shannxi, China; 9 Kunming Key Laboratory of Children Infection and Immunity, Yunnan Key Laboratory of Children’s Major Disease Research, Yunnan Institute of Pediatrics Research, Yunnan Medical Center for Pediatric Diseases, Kunming Children’s Hospital, Kunming 650228, Yunnan, China; 10 Department of Pediatric Surgery, Liuzhou Key Laboratory of Birth Defect Prevention and Control, Liuzhou Hospital, Guangzhou Women and Children’s Medical Center, Liuzhou 545616, Guangxi, China

**Keywords:** hepatoblastoma, susceptibility, TRMT61B, polymorphism, Han Chinese

## Abstract

N^1^-methyladenosine (m^1^A) is an essential chemical modification of RNA. Dysregulation of RNA m^1^A modification and m^1^A-related regulators is detected in several adult tumors. Whether aberrant RNA m^1^A modification is involved in hepatoblast carcinogenesis has not been reported. tRNA methyltransferase 61B (TRMT61B) is responsible for mitochondrial RNA m^1^A modification. Some evidence has shown that genetic variants of *TRMT61B* might contribute to cancer susceptibility; however, its roles in hepatoblastoma are unknown. This study attempted to discover novel hepatoblastoma susceptibility loci. With the TaqMan method, we examined genotypes of the *TRMT61B* rs4563180 G>C polymorphism among germline DNA samples from 313 cases and 1446 controls. The association of the rs4563180 G>C polymorphism with hepatoblastoma risk was estimated based on odds ratios (ORs) and 95% confidence intervals (CIs). We found that the *TRMT61B* rs4563180 G>C polymorphism correlated significantly with a reduction in hepatoblastoma risk (GC vs. GG: adjusted OR=0.65, 95% CI=0.49-0.85, *P*=0.002; GC/CC vs. GG: adjusted OR=0.66, 95% CI=0.51-0.85, *P*=0.002). In stratified analysis, significant associations were detected in children younger than 17 months old, girls, and subgroups with stage I+II or III+IV tumors. False-positive report probability analysis validated that children with the GC or CC genotype, particularly in those <17 months of age, had a decreased risk of hepatoblastoma. The rs4563180 G>C polymorphism also correlated with expression of *TRMT61B* and the nearby gene *PPP1CB*. We identified a high-quality biomarker measuring hepatoblastoma susceptibility, which may contribute to future screening programs.

## INTRODUCTION

Hepatoblastoma is a rare childhood malignancy in the liver, with an annual incidence of 0.5-1.5 per million children globally [1, 2]. Approximately 1.4 per million children in China develop this disease yearly [3]. Among all cases of pediatric tumors, approximately 1% are hepatoblastoma [2]. Most children with pediatric hepatoblastoma are diagnosed between 6 months and 3 years of age [1, 2]. Although the 5-year overall survival of hepatoblastoma is approximately 70%, improvements in clinical outcomes are still needed, especially for high-risk patients with much worse prognosis [4].

The etiology of hepatoblastoma remains largely unknown [2]. Apart from putative risk factors, such as prematurity, low birth weight, and parental smoking, several lines of evidence have substantiated that genetic factors contribute to hepatoblastoma susceptibility. First, several genetic syndromes have a greatly increased predisposition to hepatoblastoma, including Beckwith–Wiedemann syndrome (BWS), Simpson–Golabi–Behmel syndrome, hemihypertrophy, and trisomy 18 [5]. Affected children are recommended to undergo screening for hepatoblastoma and other pediatric malignancies [5]. Second, other groups and ours have identified some hepatoblastoma susceptibility loci in the myeloperoxidase [6], and *CCND1* [7], *xeroderma pigmentosum, complementation group C* (*XPC*) [8], *methyltransferase-like 14* (*METTL14*) [9], *METTL3* [10], *METTL1* [11], *high mobility group AT-hook 2* (*HMGA2*) [12], *tRNA (guanine-N(7)-)-methyltransferase subunit WD repeat domain 4* (*WDR4*) [13], *YTH N6-methyladenosine RNA-binding protein F1* (*YTHDF1*) [14], and *WT1-associated protein* (*WTAP*) [15] genes using the candidate gene method. Moreover, abundant functional susceptibility loci in pivotal genes warrant investigation in hepatoblastoma.

N^1^-methyladenosine (m^1^A) is one of the essential chemical modifications of RNA. Similar to RNA m^6^A modification, m^1^A is mediated by engaged and highly conserved enzymatic machinery involving methyltransferases (writers), demethylases (erasers), and YT521-B homology (YTH) domain-containing proteins (readers of methyl groups in RNAs). m^1^A is observed in tRNAs, rRNAs, mRNAs, and long noncoding RNAs (lncRNAs), with preferential enrichment in tRNAs. m^1^A may affect the processing, secondary and tertiary structures, stability, translation efficiency, and biological functions of RNAs. Dysfunction of m^1^A-associated enzymes has been known to result in cardiovascular diseases, pulmonary diseases, Alzheimer’s disease, and tumorigenesis [16, 17]. tRNA methyltransferase 6 (TRMT6) and TRMT61A are implicated in the initiation of glioma, gastrointestinal cancer, and hepatocellular carcinoma (HCC) [16, 18–22]. TRMT61B is a critical mitochondria-specific tRNA methyltransferase that is predominantly distributed in mitochondria and installs m^1^A at position 58 (m^1^A58) of tRNA (Leu (UUR), (Lys), and (Ser(UCN)), as well as 16S rRNA [23, 24]. There are very few studies regarding TRMT61B thus far. Couch et al. identified *TRMT61B* as a susceptibility gene in ER-negative breast cancer [25]. Ali et al. showed that *TRMT61B* gene variants are related to m^1^A/G RNA abundance in the mitochondria of various tissues and are consequently linked to many disease/disease-promoting conditions, such as abnormal blood pressure, breast cancer, and erythrodermic psoriasis [26]. Recently, *TRMT61B* was found to be associated with high levels of aneuploidy. *TRMT61B* knockdown leads to senescence and apoptosis of melanoma cell lines [27]. The roles of *TRMT61B* genetic variants in hepatoblastoma have not been reported. In this study, we investigated the association of a *TRMT61B* single-nucleotide polymorphism (SNP) with hepatoblastoma susceptibility in a cohort of 313 cases and 1446 healthy controls.

## RESULTS

### Association study

We successfully examined the genotype of the *TRMT61B* rs4563180 G>C polymorphism in 310 children with hepatoblastoma and 1444 healthy controls among the 313 cases and 1446 controls (Table 1). While performing univariate and multivariate logistic regression analyses, we found that the rs4563180 G>C polymorphism showed protective effects against hepatoblastoma under heterogeneous conditions [adjusted odds ratio (AOR)=0.65, 95% confidence interval (CI)=0.49-0.85, *P*=0.002]. In other words, children harboring the GC genotype showed a 35% lower risk of hepatoblastoma than those with the GG genotype. A significant association between the *TRMT61B* rs4563180 G>C polymorphism and reduced hepatoblastoma susceptibility was also found under additive (AOR=0.72, 95% CI=0.58-0.91, *P*=0.005) and dominant (GC/CC vs. GG: AOR=0.66, 95% CI=0.51-0.85, *P*=0.002) models (Table 1).

**Table 1:** Association of *TRMT61B* rs4563180 G>C polymorphism with hepatoblastoma risk.

**Genotype**	**Cases (N=310)**	**Controls (N=1444)**	***P* ^a^**	**Crude OR (95% CI)**	***P***	**Adjusted OR (95% CI) ^b^**	***P* ^b^**
rs4563180 (HWE=0.879)							
GG	213 (68.71)	853 (59.07)		1.00		1.00	
GC	83 (26.77)	515 (35.66)		**0.65 (0.49-0.85)**	**0.002**	**0.65 (0.49-0.85)**	**0.002**
CC	14 (4.52)	76 (5.26)		0.74 (0.41-1.33)	0.312	0.74 (0.41-1.33)	0.316
Additive			0.005	**0.73 (0.58-0.91)**	**0.005**	**0.73 (0.58-0.91)**	**0.005**
Dominant	97 (31.29)	591 (40.93)	0.002	**0.66 (0.51-0.85)**	**0.002**	**0.66 (0.51-0.85)**	**0.002**
GG/GC	296 (95.48)	1368 (94.74)		1.00		1.00	
CC	14 (4.52)	76 (5.26)	0.589	0.85 (0.48-1.53)	0.589	0.85 (0.48-1.53)	0.597

OR, odds ratio; CI, confidence interval; HWE, Hardy-Weinberg equilibrium.

^a^χ^2^ test for genotype distributions between hepatoblastoma patients and controls.

^b^Adjusted for age and gender.

### Stratified analysis

Stratified analysis revealed significant associations in children younger than 17 months old (AOR=0.62, 95% CI=0.43-0.89, *P*=0.010), girls (AOR=0.57, 95% CI=0.38-0.86, *P*=0.008), and those with hepatoblastoma in stages I+II (AOR=0.65, 95% CI=0.46-0.93, *P*=0.018) or III+IV (AOR=0.55, 95% CI=0.34-0.88, *P*=0.012) (Table 2).

**Table 2:** Stratification analysis for the association between *TRMT61B* rs4563180 genotypes and hepatoblastoma risk.

**Variables**	**GG**	**GC/CC**	**Crude OR**	***P***	**Adjusted OR ^a^**	***P* ^a^**
	**(Cases/Controls)**		**(95% CI)**		**(95% CI)**	
Age, month						
<17	115/369	52/272	**0.61 (0.43-0.88)**	**0.008**	**0.62 (0.43-0.89)**	**0.010**
≥17	98/484	45/319	0.70 (0.48-1.02)	0.063	0.70 (0.48-1.02)	0.066
Gender						
Females	91/347	37/248	**0.57 (0.38-0.86)**	**0.008**	**0.57 (0.38-0.86)**	**0.008**
Males	122/506	60/343	0.73 (0.52-1.02)	0.063	0.73 (0.52-1.02)	0.063
Clinical stage						
I+II	110/853	50/591	**0.66 (0.46-0.93)**	**0.018**	**0.65 (0.46-0.93)**	**0.018**
III+IV	66/853	25/591	**0.55 (0.34-0.88)**	**0.012**	**0.55 (0.34-0.88)**	**0.012**

OR, odds ratio; CI, confidence interval.

^a^Adjusted for age and gender, omitting the corresponding stratify factor.

### FPRP analysis

We also conducted false-positive report probability (FPRP) analyses for significant findings (Table 3). The prior possibility is referred to as the possibility that the association of an SNP with a disease is genuine. Our results indicated that with a high prior possibility of 0.25, all significant associations were deserving of attention. Next, when we adopted a moderate prior possibility of 0.1, the association of the *TRMT61B* rs4563180 G>C polymorphism with hepatoblastoma susceptibility remained trustworthy in the overall analysis and among girls in stratified analysis (Table 3). These results suggest that children with the GC or CC genotype have a reduced risk of hepatoblastoma, especially girls.

**Table 3:** False-positive report probability analysis for significant findings derived from *TRMT61B* rs4563180 and hepatoblastoma risk.

**Genotype**	**OR (95% CI)**	***P* ^a^**	**Statistical power ^b^**	**Prior probability**				
				**0.25**	**0.1**	**0.01**	**0.001**	**0.0001**
GC vs. GG	0.65 (0.49-0.85)	0.002	0.411	**0.014**	**0.040**	0.314	0.822	0.979
GC/CC vs. GG	0.66 (0.51-0.85)	0.002	0.450	**0.011**	**0.033**	0.272	0.791	0.974
<17 months	0.61 (0.43-0.88)	0.008	0.326	**0.072**	**0.188**	0.719	0.963	0.996
Female	0.57 (0.38-0.86)	0.008	0.230	**0.092**	0.234	0.770	0.971	0.997
Stage I+II	0.66 (0.46-0.93)	0.018	0.459	**0.107**	0.265	0.799	0.976	0.998
Stage III+IV	0.55 (0.34-0.88)	0.012	0.210	**0.148**	0.343	0.852	0.983	0.998

OR, odds ratio; CI, confidence interval.

^a^Chi-square test was used to calculate the genotype frequency distributions.

^b^Statistical power was calculated using the number of observations in each subgroup and the corresponding ORs and *P* values in this table.

### Expression quantitative trait locus (eQTL) analysis

**Figure 1:**
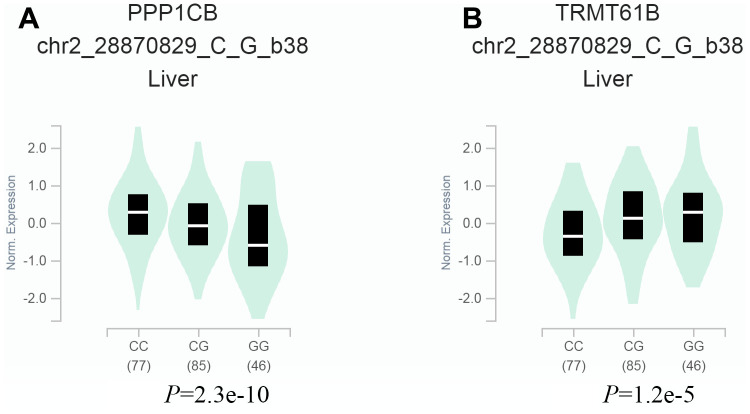
**Relationship between the *TRMT61B* rs4563180 G>C polymorphism and gene expression.** (**A**, **B**) Gene Tissue Expression (GTEx) analysis indicated downregulation of the nearby protein phosphatase 1 catalytic subunit beta (*PPP1CB*) gene and upregulation of the *TRMT61B* gene in liver tissues with the GG genotype compared to the CC genotype.

We explored the potential effects of the *TRMT61B* rs4563180 G>C variant on the expression of *TRMT61B* and neighboring genes. Our results indicated significantly decreased nearby *protein phosphatase 1 catalytic subunit beta* (*PPP1CB*) gene expression (*P*=2.3e-10) (Figure 1A) and an increase in that of the *TRMT61B* gene (*P*=1.2e-10) in livers with the GG genotype compared with CC genotype (Figure 1B). These results suggest that the *TRMT61B* rs4563180 G>C polymorphism may affect expression of essential genes.

## DISCUSSION

Hepatoblastoma, especially the high-risk subtype, is rare but devastating. Genetic background plays a vital role in the initiation of hepatoblastoma. Therefore, some genetic syndromes are known to predispose patients to hepatoblastoma. For affected children, routine screening every three months is an effective strategy for early diagnosis of this disease. However, there is a lack of reliable genetic susceptibility biomarkers for large-scale screening in the community [5]. Early diagnosis and effective treatment are crucial to improving childhood cancer outcomes. It is urgent to uncover more hepatoblastoma-predisposing loci.

Large case-control studies coupled with candidate gene methodology have facilitated discovery of numerous disease susceptibility genes in various disorders. However, such studies in hepatoblastoma are minimal due to its low incidence. Most case-control studies contain fewer than 100 samples from hepatoblastoma patients [6, 7]. Fortunately, we have established a relatively large cohort of 313 cases and 1446 controls over the past years. Our group has previously reported that many genetic variants of genes encoding RNA m^6^A and m^7^G methyltransferase, demethylase, and m^6^A-reading proteins confer hepatoblastoma susceptibility, including *METTL3* [10], *METTL4* [9], *AlkB homolog 5* (*ALKBH5*) [28], *FTO* [29], *YTHDC1* [30], *YTHDF1* [14], *WTAP* [15], *WDR4* [13], and *METTL1* [11].

m^1^A is also one of the essential reversible chemical modifications of RNA and is installed on the first nitrogen atom of adenosine [17]. m^1^A modifications are much less prevalent in mRNA than m^6^A but preferentially occur in tRNA. Many studies have implicated RNA m^1^A in cardiovascular diseases, pulmonary diseases, Alzheimer’s disease, and tumorigenesis [16, 17]. In HCC, m^1^A modification levels in tRNA are remarkably upregulated, which was attributed to the highly expressed m1A methyltransferase complex TRMT6/TRMT61A [22]. TRMT6/TRMT61A promotes PPARδ translation by specifically enhancing m^1^A methylation in relevant tRNAs, further activating hedgehog signaling and stimulating self-renewal of liver CSCs and tumorigenesis [22]. Ye et al. confirmed TRMT6 overexpression in HCC tissues, and ectopic expression of TRMT6 accelerates HCC cell proliferation and stimulates cell cycle progression by activating the PI3K/AKT pathway [19]. TRMT6/61A overexpression was also observed in urothelial carcinoma of the bladder, along with dysregulation of the tRF targetome. TRMT6/61A-induced upregulated m^1^A modification on tRFs plays an essential role in maintaining a proper unfolded protein response [18].

*TRMT61B* is a crucial tRNA methyltransferase in mitochondria, which has a genome composed of 2 rRNA genes, 22 tRNA genes, and 13 mRNA genes. m^1^A RNA modification levels affect mitochondrial transcript processing. Genetic variants of *TRMT61B* have been shown to associate with mitochondrial m^1^A RNA modification degrees at functionally crucial sites in a broad spectrum of tissues [26]. These results implied that *TRMT61B* variants might have a profound impact on the function of mitochondrial RNAs. Coincidently, Couch et al. determined that *TRMT61B* at 2p23.2 is a susceptibility gene in ER-negative breast cancer based on functional and eQTL studies [25]. Recently, *TRMT61B* was found to be associated with high levels of aneuploidy, and its knockdown led to senescence and apoptosis of melanoma cell lines [27].

Given the importance of m^1^A in tumorigenesis and the impacts of *TRMT61B* gene variants on m^1^A RNA levels, it is necessary to explore the association between *TRMT61B* gene SNPs and hepatoblastoma susceptibility. In this study, we found that the *TRMT61B* rs4563180 G>C polymorphism was significantly associated with a decreased risk of hepatoblastoma. Significant associations were found for children <17 months of age, girls, and subgroups with stage I+II or III+IV disease in stratified analysis. It should be noted that genetic association studies generally require many statistical tests. In such cases, significant associations between genetic variants and susceptibility to diseases based on *P* values may only be spurious findings. Therefore, PFRP analyses were performed to validate our significant findings by integrating the prior possibility, study power, and *P* values. With a moderate prior possibility of 0.1, PFRP analyses indicated that the association of the *TRMT61B* rs4563180 G>C variant with the risk of hepatoblastoma was trustworthy in the whole population and in girls. Interestingly, GTEx analyses revealed that the *TRMT61B* rs4563180 G>C variant might modify expression of the *PPP1CB* and *TRMT61B* genes. The protein product of the *PPP1CB* gene is one of the three catalytic subunits of protein phosphatase 1 (PP1), which is a serine/threonine protein phosphatase regulating many fundamental cellular processes (e.g., cell division and protein synthesis) [31]. Recently, a genetic variant of PPP1CB was reported to modify the risk of hepatitis B virus-related HCC [32]. Collectively, we identified *TRMT61B* as a high-quality hepatoblastoma gene, and the roles of its genetic variant in disease susceptibility deserve further attention.

Attention should also be given to the limitations of this study. First, we made great efforts to recruit subjects from seven independent medical centers across China. However, the sample size was moderate because of the disease’s rareness, which limited the study’s statistical power, especially with regard to stratified analysis. Second, the study population was restricted to Han Chinese ethnicity. Third, potential environmental risk factors were not included in analyses because of the inaccessibility of the information. Fourth, we could not perform survival analysis without relevant information. Finally, only one SNP that met the selection criteria was investigated in this study.

In summary, we discovered that the *TRMT61B* rs4563180 G>C variant significantly reduced the risk of childhood hepatoblastoma. This SNP may be used to screen for children at high risk of hepatoblastoma when combined with other essential susceptibility loci.

## MATERIALS AND METHODS

### Study population

Complete information on the cohort was provided in previous publications [15]. In brief, 313 children with hepatoblastoma and 1446 healthy controls (Supplementary Table 1) of Han Chinese nationality were recruited from participating medical centers located in different cities across China, including Taiyuan, Guangzhou, Kunming, Zhengzhou, Changsha, Xi’an, and Shenyang. All patients were newly diagnosed and confirmed by two or more pathologists. The clinical stages of the patients were determined using the PRETEXT classification [33]. Healthy volunteers were recruited from the same seven medical centers above who visited for routine examinations. Epidemiological and clinical data on these children were reported previously [15]. We obtained signed informed consent for every subject from their parents or guardians.

### Identification and genotyping of SNPs

Following standard criteria [34, 35], we determined the rs4563180 G>C polymorphism as a potential functional SNP in the *TRMT61B* gene. We predicted SNP functions using the online tool SNPinfo (https://snpinfo.niehs.nih.gov/snpinfo/snpfunc.html). The results retrieved showed that the rs4563180 polymorphism is located in the transcription factor-binding site (TFBS). Therefore, this SNP may affect binding of transcription factors to the *TRMT61B* gene, consequently modulating its transcription. DNA was extracted from peripheral blood samples of the participants with Tiangen Blood DNA Extraction Kit (Taigen Biotechnology, Beijing, China). Genotypes of samples were examined using the TaqMan instrument (Applied Biosystems, Foster City, CA, USA) and a 384-well plate format. Positive and negative control samples were run in parallel with the samples in each assay to ensure genotyping quality. Laboratory workers who performed the experiments were blinded to the identities of the specimens. Routinely, a fixed fraction of DNA samples was randomly selected for validation testing. A concordance rate of 100% for the repeated tests was needed.

### Genotype-tissue expression (GTEx) analysis

The GTEx project was initiated by the National Institutes of Health in September 2010. This project generated a database that allows researchers to study relationship between inherited gene changes and common diseases. This resource contains expression of genes in various tissues from many different people, accompanied by genotypes of numerous SNPs [36]. This web tool was used to explore the impacts of the rs4563180 G>C polymorphism on gene expression.

### False-positive report probability analysis

Genetic association studies are usually subjected to many statistical tests, with statistical significance determined by *P* values. As a result, some associations between genetic variants and diseases based on a *P* value below 0.05 alone may be falsely positive. To eliminate false significant associations between genetic variants and hepatoblastoma, FPRP analysis was adopted by considering the prior probability of a genuine association of the SNPs with a disorder, the power of statistics, and *P* values. Prior probabilities of 0.1, 0.01, or 0.001 were defined as high, moderate, or low, respectively. A given association with an FPRP value below 0.2 deserves attention or trustworthiness [37, 38].

### Statistical analysis

A t test or χ^2^ test was applied to check the significant differences between the cases and controls for continuous or categorical variables, respectively. A goodness-of-fit χ^2^ test was employed to evaluate the Hardy-Weinberg equilibrium (HWE) of the tested SNP in the controls. Finally, the statistical significance of the association of the *TRMT61B* SNP and hepatoblastoma susceptibility was determined using unconditional logistic regression analysis. The strength of the association between the *TRMT61B* SNP and hepatoblastoma susceptibility was measured by ORs and 95% CIs. Stratified analyses by age, sex, and clinical stage were carried out. A two-sided *P*<0.05 was accepted as statistically significant. All analyses were completed with SAS v9.4 (SAS Institute Inc., Cary, NC, USA).

### Data availability statement

All the data are available upon request.

## Supplementary Material

Supplementary Table 1
