# Supplementary material for: TRMT61B rs4563180 G>C variant reduces hepatoblastoma risk: a case-control study of seven medical centers
Source: Aging (Albany NY). 2023 Aug 1;15(15):7583–92. doi: 10.18632/aging.204926 (PMC10457066; doi:10.18632/aging.204926)
Supplement: Supplementary Table 1 [file aging-15-204926-s001.pdf]

SUPPLEMENTARY TABLE

Supplementary Table 1. Frequency distribution of selected variables in hepatoblastoma patients and cancer-free controls.

Variables	Cases (n=313)		Controls (n=1446)		P^a
	No.	%	No.	%	
Age range, month	0.03-149.97		0.004-156.00		
Mean ± SD	23.75 ± 25.93		25.23 ± 19.38		0.251[b]
<17	168	53.67	642	44.40	
≥17	145	46.33	804	55.60	
Gender					0.983
Female	129	41.21	595	41.15	
Male	184	58.79	851	58.85	
Clinical stages					
I	97	30.99	/	/	
II	63	20.13	/	/	
III	64	20.45	/	/	
IV	27	8.63	/	/	
NA	62	19.81	/	/	

SD, standard deviation; NA, not available.

[a]Two-sided χ^2 test for distributions between hepatoblastoma cases and cancer-free controls.

[b]T-test for age distribution between hepatoblastoma patients and cancer-free controls.
